# Biosynthesis, classification, properties, and applications of *Weissella* bacteriocins

**DOI:** 10.3389/fmicb.2024.1406904

**Published:** 2024-06-12

**Authors:** Jahnavi Kumari Singh, Palanisamy Bruntha Devi, G. Bhanuprakash Reddy, Amit K. Jaiswal, Digambar Kavitake, Prathapkumar Halady Shetty

**Affiliations:** ^1^Department of Food Science and Technology, Pondicherry University, Pondicherry, India; ^2^Biochemistry Division, Indian Council of Medical Research (ICMR)-National Institute of Nutrition, Hyderabad, Telangana, India; ^3^School of Food Science and Environmental Health, Faculty of Sciences and Health, Technological University Dublin, Dublin, Ireland

**Keywords:** *Weissella* genus, *Weissella* bacteriocins, biosynthesis, properties, food and health applications

## Abstract

This review aims to comprehensively chronicle the biosynthesis, classification, properties, and applications of bacteriocins produced by *Weissella* genus strains, particularly emphasizing their potential benefits in food preservation, human health, and animal productivity. Lactic Acid Bacteria (LAB) are a class of microorganisms well-known for their beneficial role in food fermentation, probiotics, and human health. A notable property of LAB is that they can synthesize antimicrobial peptides known as bacteriocins that exhibit antimicrobial action against both closely related and other bacteria as well. Bacteriocins produced by *Weissella* spp. are known to exhibit antimicrobial activity against several pathogenic bacteria including food spoilage species, making them highly invaluable for potential application in food preservation and food safety. Importantly, they provide significant health benefits to humans, including combating infections, reducing inflammation, and modulating the gut microbiota. In addition to their applications in food fermentation and probiotics, *Weissella* bacteriocins show promising prospects in poultry production, processing, and improving animal productivity. Future research should explore the utilization of *Weissella* bacteriocins in innovative food safety measures and medical applications, emphasizing their potential to combat antibiotic-resistant pathogens, enhance gut microbiota composition and function, and synergize with existing antimicrobial therapies.

## Graphical Abstract

Schematic representation of Weissella bacteriocin production, classification and applications. 
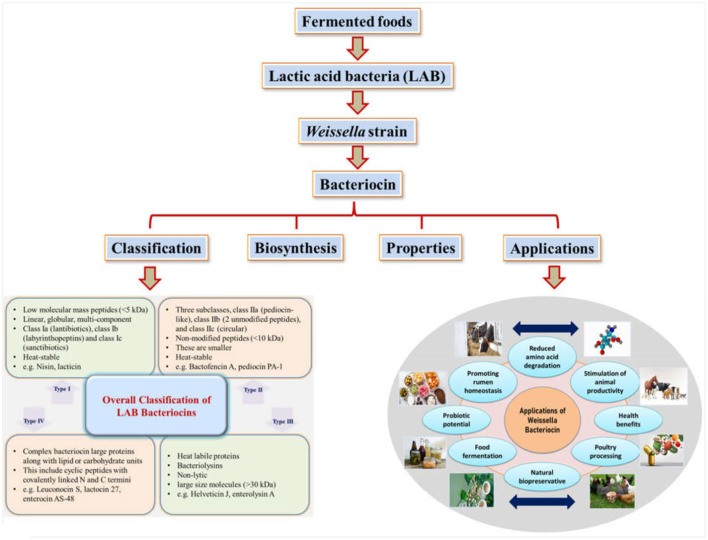

## 1 Introduction

Bacteriocins are bioactive peptides or proteins produced through ribosomal synthesis by bacteria. They are recognized for their ability to hinder the growth of closely related bacterial species, which is why they are referred to as bacteriocins (Daw and Falkiner, 1996). Several bacteria produce these antimicrobial peptides as part of their survival strategy. Depending on their size and mode of action, bacteriocins can either be lethal to bacteria (bactericidal) or inhibit their growth (bacteriostatic) (Nes et al., 2007). Bacteriocins mostly possess anti-microbial action either by inducing pore formation in the cell wall of the target organism or inhibiting cell wall biosynthesis, consequently resulting in death (Zacharof and Lovitt, 2012; Malik et al., 2021). The immunity of bacteriocin-producing organisms against their bacteriocins is facilitated by immunity proteins (Cotter et al., 2005). According to the reported studies (Klaenhammer, 1993; Kotelnikova and Gelfand, 2002; Chavan and Riley, 2007), bacteriocin synthesis typically occurs during the early stationary or late exponential phases of development. It is also commonly regulated by stress signaling and quorum sensing mechanisms. It is regarded as an adaptive probiotic trait causing the clampdown of gut-based pathogens (Dobson et al., 2012; O'Shea et al., 2012).

Antimicrobial peptides have gained interest recently as novel antimicrobials to fight dangerous bacteria, particularly those resistant to traditional antibiotics. Such bioactive substances might serve as models for creating new entities in the hunt for new bio-preservatives or drugs (Goh and Philip, 2015). Earlier reports have revealed the diverse capabilities of LAB isolates including probiotic potential, pathogen growth inhibition, mycotoxin degradation (Naidu et al., 1999; Todorov et al., 2020). Additionally, the rising interest in their use as natural preservatives for preserving foods has been fueled by the surge in demand for natural foods free of chemical preservatives (Gupta and Tiwari, 2014). Many bacteriocins produced by *Lactobacillus, Enterococcus* and *Leuconostoc* species have already been studied earlier (Yang et al., 2018; Cherukuri et al., 2019). However, the information on bacteriocins from *Weissella* species is limited and can be investigated further for possible applications (Cleveland et al., 2001). Additionally, some *Weissella* strains were reported to produce exopolysaccharides (EPS), which have different techno-functional and biological properties including prebiotic capabilities (Kavitake et al., 2016, 2020; Devi et al., 2021; Teixeira et al., 2021).

*Weissella* genus has consequently been acknowledged for its remarkable ability to endure the gastrointestinal tract (GIT), producing antimicrobial substances effective against numerous pathogens, and promoting the production of compounds that improve the gut microbiome. This review aims at providing up to date information on bacteriocins derived from the *Weissella* genus. It discusses production, classification, physico-chemical and functional properties, inhibitory mechanisms, and their diverse applications of bacteriocins from *Weissella* across various fields.

## 2 Bacteriocin biosynthesis

Bacteriocin production, control, self-immunity, transport, and modification are all regulated by the biosynthetic genes known as bacteriocin genes (Sahl and Bierbaum, 1998; McAuliffe et al., 2001; Dimov et al., 2005). The fundamental route involves the creation of pre-bacteriocin and further separation of the pre-peptide at a particular processing site, eliminating the leader sequence and leading translocation of pro-bacteriocin outside the cell membrane (Abdulkarim et al., 2020; Simons et al., 2020). Various genes play a vital role in bacteriocin biosynthesis processes (Table 1). These genes are generally organized as operons which can be found on conjugative transposable elements (nisin), over the host chromosomes (subtilin), and on the plasmids (cytolysin) (Banerjee and Hansen, 1988; Ike et al., 1990; Immonen and Saris, 1998). For instance, in the synthesis of most common bacteriocins, structural genes are responsible for the encoding of lantibiotics, which are usually referred to as *lan*, which denotes lantibiotics and the latter alphabet denotes functional aspect. This configuration of minimum genetic machinery includes genes required for modification (*lan*M), ABC-type peptide translocators (*lan*T), proteolytic processing (*lan*P), immunity (*lan*I), and regulation (*lan*R) (Klaenhammer, 1993; McAuliffe et al., 2001). The entire mechanism of bacteriocin production and release is based on signal transduction systems, where the general secretory path (GSP) and the signal peptide type sequence (SP) are crucial in processing and releasing bacteriocins (Kotelnikova and Gelfand, 2002). There are three major components, the inductor peptide (IP), response regulatory protein (RR), and sensor Histidine Protein Kinase (HPK). Moreover, genes with related functions are in close proximity to the cluster (Li et al., 2017).

**Table 1:** Vital genetic components of bacteriocin biosynthesis process.

**Genes involved in bacteriocin biosynthesis**	**Genes**	**Properties/function**
Structural genes	C-39 peptidase domain with ABC transporter (Gly-Gly leader motif) MDKLSKFESLSDANLSTIVG (Leader sequence) GSDKNNVFFQIGKRYVAPVLYWFGKGAEGIKG (Core peptide) *Sec* signal peptides *welY* and *welM* (42 AND 43 amino acids)	Encode the pre-pro-bacteriocin, having an N-terminal (the leader sequence is exclusively double glycine type or peptide signal type sequence). The presence of two conserved glycines at its C-terminus, further recognized by ABC transporters to process the leader sequence helping in extracellular secretion of the mature bacteriocin
Immunity genes	*Abi* gene (CAAX motif)	Small proteins consisting of 51–154 amino acids. Their purpose includes protection of the bacteriocin producing strain.
Genes encoding proteins	Secretory accessory protein	Responsible for processing, transport and secretion of the pre-probacteriocin.
Modification genes	ATP-binding protein ComA Permease protein ComB	Encode the enzyme causing post-translational modifications of the probacteriocin.
Regulatory genes	Cellobiose-specific IIC component Ribosome Binding Sites (RBS)	Encode the genes involved in the regulation of bacteriocin synthesis.

References: Ennahar et al. (2000); Field et al. (2007); Nes et al. (2007); Masuda et al. (2012); Li et al. (2017); Noda et al. (2018); Todorov et al. (2020).

## 3 Mode of action and resistance

Bacteriocins have the ability to operate as signaling, killing, and colonizing peptides. Cell-to-cell contact between members of the same or different species may arise from the interaction with the GI tract, enabling both cooperative and hostile microbial interactions (Perez et al., 2018). Furthermore, mechanistic activity of enterocin B and enterocin ST5Ha against H3N2, H1N138, and HSV type 137 respectively was regarded similar to class II bacteriocins. Moreover, bacteriocin Subtilosin A does not function prior to HSV type 1 and 2 viral protein synthesis at lower concentrations but has a virucidal action toward assembly or release of concerned proteins (Elalem, 2021).

Studies predict bacteriocin enters the target cell via cell surface receptors and creates ion-permeable channels in the cytoplasmic membrane, degrading cellular DNA non-specifically. This consequently inhibits the synthesis of proteins and peptidoglycan by specifically cleaving 16s rRNA, or causing cell lysis (Riley and Wertz, 2002). Similarly, a bacteriocin named Plantaricin CYLB47 targets *Staphylococcus aureus* and *Pseudomonas aeruginosa* by the mechanism of action of lantibiotics (type A lantibiotics that kill byforming pores, or type B lantibiotics that impede the biosynthesis of peptidoglycans) (Thuy et al., 2024). Limited studies mention an in-depth information with regard to few lactic acid bacteria, their gene structures, routes, and mechanisms of action of Class I, II, III, and IV bacteriocins from different ethnic background (Sharma et al., 2021). Specific to *Weissella* strains, bacteriocins 7293A, and B plausibly worked on the cytoplasmic membrane of target cells by creating hydrophilic portions that allowed essential cellular molecules to be effluxed and ultimately led to cell death (Papagianni and Papamichael, 2011; Woraprayote et al., 2015).

Target organisms naturally have a tendency for an environmental adaptation. After prolonged exposure, they acquire components that make them resistant to bacteriocins. Few ways of resistance development are, modifications in the fatty acid composition of the membrane; increase in the d-alanine content of teichoic acid wall; increase in the l-lysine content and modification of phospholipids and lastly changes in the structural genetic elements. By combining the bacteriocin with other antimicrobial substances like essential oil and organic acids and peptides increased the susceptibility of the target organisms. Other methods for effective outcomes include encapsulation, irradiation, and synergistic combinations of bacteriocins (Skaugen et al., 2003). One promising area of bioengineering has been the rational design of bacteriocins to enhance their functionality. Modification at selected residues of bactofencin and lacticin 3147 A increased their antibacterial efficacy (Kumariya et al., 2019).

## 4 Bacteriocins from LAB

LABs are gram-positive, non-aerobic and non-spore forming but aerotolerant cocci or cocco-bacilli bacteria. These are known for lactic acid production by fermenting carbohydrates. Belonging to the firmicutes phylum, they include most bacteria having probiotic properties (Liptáková et al., 2017; Bintsis, 2018). LABs are found ubiquitously in environments of milk, meat, fermented vegetables, and beverages. They were first discovered in milk, soil, water, manure, and sewage (Liu et al., 2014). Humans and animals also harbor LAB which has different functional roles such as inhibition of pathogen growth, extending the shelf life of foods, and enhancing the nutritive qualities of the product (Martinez et al., 2013). They have also been used as enhancing agents to improve flavor and texture (Silva et al., 2018). Some LAB like *Streptococcus thermophilus* and *Lactobacillus lactis*, possess the inhibiting capability for pathogenic bacterial growth, avoid food spoilage along with the simultaneous advantage of preserving the nutritive qualities of food material consequently giving it an extended shelf life. Additionally, they tend to produce growth-inhibitory substances like bacteriocins, hydrogen peroxide, diacyls, and others. Based on the type of products produced after the fermentation of carbohydrates, LAB can be classified into two types: homofermentative and heterofermentative microorganisms. Homofermentative LAB chiefly utilizes sugars to bring forth lactic acid, while heterofermentative produces lactic acid, alcohols, acetic acid, and carbon dioxide (Riley and Wertz, 2002; Teixeira et al., 2021). Additionally, LAB is better known for the production of bacteriocins of various kinds; a few of them are mentioned in Table 2.

**Table 2:** List of few bacteriocins produced by LAB.

**Producing strain**	**Bacteriocin**	**Source**	**References**
*Lactobacillus* *johnsonii*	Lactacin F	Fermented foods and the oral, vaginal, and gastrointestinal tracts of humans and animals	Klaenhammer et al., 1994
*Lactococcus lactis*	Lactococcin Q	Corn	Zendo et al., 2006
*Lactococcus lactis* subsp. lactis NIZO 221 86	Nisin Z	Dairy foods and Human gut	Mulders et al., 1991
*Leuconostoc* spp.	Leucocin H	Cereals, Sea foods, Dairy foods, Vegetables and fruits	Blom et al., 1999
	Leucocin C-607	Persimmon	Chen et al., 2018
	Leucocin B-Ta11a	Meat	Etayash et al., 2014
*Lactiplantibacillus plantarum*	Plantaricin 423	Sorghum beer	Reenen, 1998
	Plantaricin W	Tempoyak-Indonesian Fermented Food	Holo et al., 2001
	Plantaricin C	Dairy origin	Gonzalez et al., 1994
*Pediococcusacidilactici* PAC-1.0	Pediocin NV 5	Vacuum-Packed Fermented Meat	Mandal et al., 2011
*Pediococcus acidilactici* LAB 5	Pediocin PA-1	Fermented foods	Rodríguez et al., 2002

The properties of LAB including synthesis of bacteriocins make it quite applicable as a natural preservative in food-based industries (Mokoena, 2017). Bacteriocin-producing LAB has been exploited as bio-preservatives and has contributed immensely to a diversity of food fermentations (Malik et al., 2021). A significant portion of bacteriocin research has revolved around the synthesis and examination of peptides derived out of various LAB species, *including Lactococcus* spp*., Leuconostoc* spp., *Enterococcus* spp., and *Pediococcus* spp. to explore the prospected use of these peptides as naturally originated food preservatives (Deegan et al., 2006; Chavan and Riley, 2007; Cheikhyoussef et al., 2009). Pediocin CP2, a natural antimicrobial peptide produced by *Pediococcus acidilactici* MTCC 5101 was studied for cytotoxicity toward the following cell lines: Sp2/0-Ag14 (a spleen lymphoblast), MCF7 (a mammary gland cancer), HeLa (a cervical adenocarcinoma), and HepG2 (a hepatocarcinoma cell line). The MTT and DNA fragmentation assays demonstarted the anti-cancer property of its recombinant version (Kumar et al., 2012). Bovicin HC5, which is produced by *Streptococcus bovis* HC5, was tested for its cytotoxic effects on following eukaryotic cell lines: HepG2 (a human liver hepatocellular carcinoma cell line), MCF-7 (a human breast adenocarcinoma cell line), and Vero cells (a monkey kidney epithelial cell line) (Paiva et al., 2012). AS-48 bacteriocin, obtained from *Enterococcus faecalis* strain UGRA10 has already become a commercial option for pharmacological development to prevent and cure infections, including those caused by multidrug-resistant microorganisms, especially in the skin and soft tissues (Cebrián et al., 2019).

## 5 Classification and nomenclature of bacteriocins

**Figure 1:**
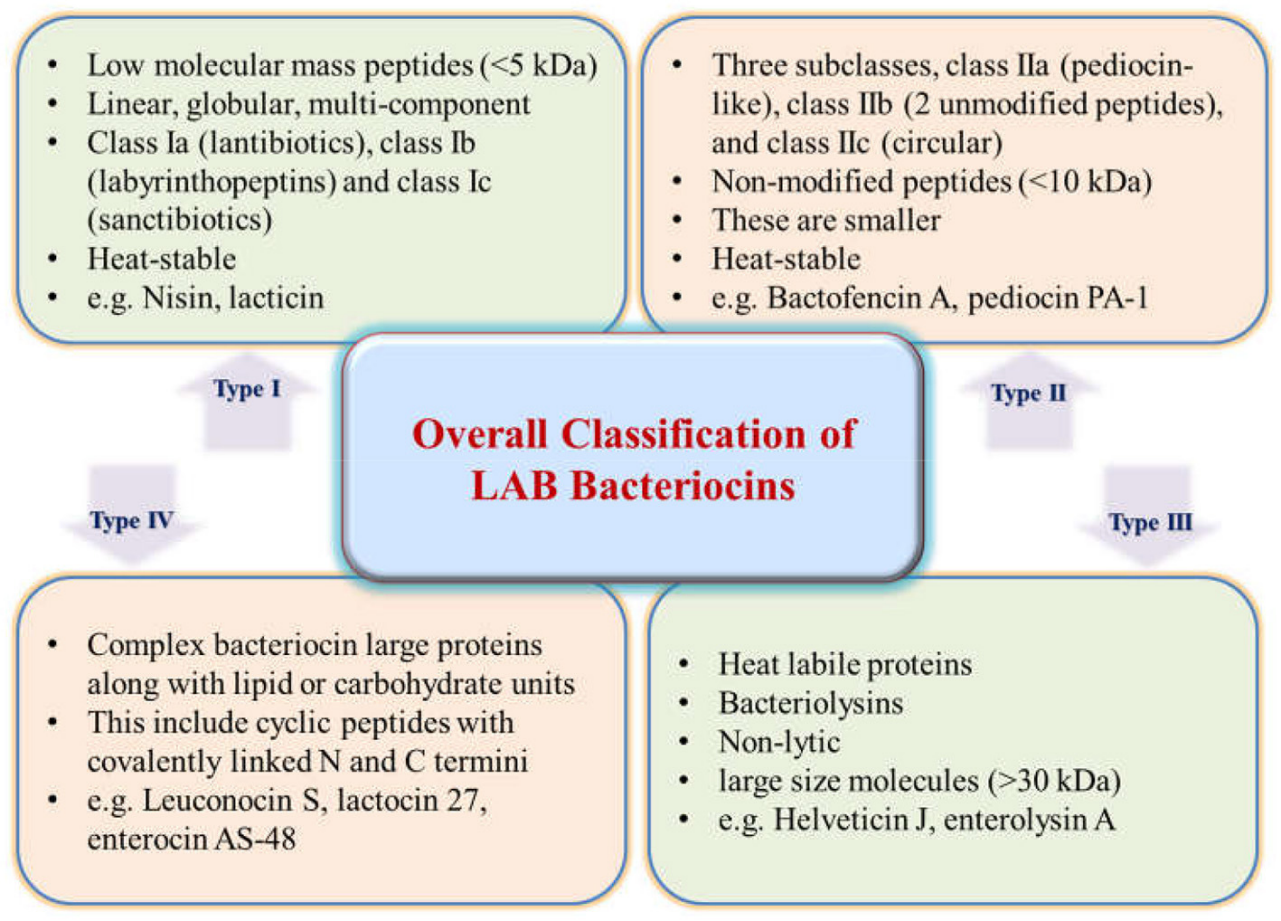
Classification of bacteriocins.

The initial scheme of classification grew with new bacteriocins being identified and characterized. Eventually, based on the structural transformations, bacteriocins were categorized under new sub-classes (Montville and Kaiser, 1993; Kaiser, 2014; Zimina et al., 2020). Currently, four main classes have been figured out and recognized after reviewing the literature for several years (Figure 1). Lantibiotics, also called Class I bacteriocins, are a group of peptides with low molecular mass (< 5 kDa) that exhibit heat stability. These peptides are differentiated by the constitution of specific amino acids like lanthionine or β-methyl lanthionine that are the result of post-translational modifications (Ng et al., 2020). The Class I category can be classified into three more subgroups: Class Ia (lantibiotics), Class Ib (labyrinthopeptins), and Class Ic (sanctibiotics). Among these, nisin is the most studied and established bacteriocin belonging to the Class I category (Cuozzo et al., 2001; LeLay et al., 2016). Class II bacteriocins are non-modified smaller peptides (< 10 kDa) that are thermo-stable. They are divided into further subclasses: Class IIa, known as pediocin-like; Class IIb, consisting of two-chain peptides; and Class IIc, which includes single-chain peptides that are non-pediocin-like (Yang et al., 2018). The class II bacteriocins in the LAB, sakacin A (SakA), pediocin PA-1 (PedPA-1), enterocins P, Q, and L50 (enterocins), plantaricins EF and JK (plantaricins), and garvicin ML (GarML), differ significantly from one another in terms of their physicochemical characteristics and range of inhibition. They are also known to have a potential role in modifying gut microbiota to improve host health (Umu et al., 2016). On the other hand, Class III bacteriocins include larger proteins (>30 kDa) that are thermo-labile. Finally, Class IV bacteriocins encompass cyclic peptides with N and C termini that are covalently linked (Meade et al., 2020).

Bacteriocins initially have a very low molecular weight, but eventually, they go through post-translational modifications and thus are susceptible to proteolytic enzymes (Lajis, 2020). In general, they are abundantly constituted of lysyl and arginyl residues, making them amphipathic molecules by nature. Structural studies reveal that they are unstructured in aqueous solutions but once coming in exposure to structure favoring solvents (e.g., anionic phospholipid membranes or trifluoroethanol), they form a helical structure (Zacharof and Lovitt, 2012). Bacteriocins are typically titled after the species or genus of the producer strain. For instance, strains of *Lactiplantibacillus plantarum* (formerly *Lactobacillus plantarum*) are known to produce plantaricin, *Lactococcus* spp. produce lacticin and nisin, *Carnobacterium* spp. produce carnocin, *Enterococcus* spp. produce enterocin, *Leuconostoc* spp. produce leucocin, and *Pediococcus* spp. produce pediocin. These naming conventions help identify and differentiate the bacteriocins produced by specific bacterial strains (O'sullivan et al., 2002; Zouhir et al., 2010; Zheng et al., 2020).

There exists a distinct category of bacteriocins known as high molecular weight (HMW) bacteriocins, have been observed to have a structure like that of phage tails. The resemblance between HMW bacteriocins and bacteriophages has been accepted by examining their morphological characteristics through electron microscopy, immunological cross-reactivity, complementing properties, and DNA hybridization traits (Klaenhammer, 1993).

## 6 Bacteriocins from *Weissella* genus

*Weissella* spp. comes under Gram-positive, non-motile, catalase-negative, and non-sporing group bacteria. Currently, the *Weissella* genus encompasses approximately 22 officially recognized species and most of these species are obtained from widely screened sources, with a particular emphasis on fermented foods (Kavitake et al., 2016, 2020). As members of LAB, *Weissella* spp. has specific growth requirements due to their natural habitat in environments rich in nutrients. They can be found in various sources such as vegetables, raw milk fish, meat, sewage, soil, blood, human and animal gastrointestinal tract, human oral cavity, and urogenital tract (Abriouel et al., 2015). Few *Weissella* spp. have been reported to be a cause of disease occurrences like sepsis, otitis, endocarditis and fish mortality (Harlan et al., 2011; Welch and Good, 2013; Kamboj et al., 2015). Infections in the human population resulting from *Weissella* spp. are infrequently documented, typically occurring for individuals who are susceptible to infections (Lee et al., 2012). Notably, the *Weissella* genus comprises both beneficial and harmful strains. However, it also plays a significant part in food fermentation and is identified as a potent probiotic culture with numerous health benefits (Fusco et al., 2015). *Weissella* spp. is commonly found in naturally fermented foods and actively contributes to the unique qualities of fermented products. These bacteria possess numerous technological and functional properties that have the potential to improve the safety, nutritional worth, and sensory construct of food (Fusco et al., 2018).

In addition to their recognized role through traditional fermentations, certain strains of *Weissella* spp. are gaining attention as putative probiotics. Specifically, the development of *W. cibaria* strains is being explored due to their significant probiotic potential in managing periodontal disease (Fusco et al., 2015). Despite the characterization of numerous strains, only a few bacteriocins from *Weissella* spp. have been identified, as outlined in Table 3. Furthermore, there are scarce reports on bacteriocin production by the commonly encountered *Weissella confusa* (Goh and Philip, 2015). It was not until 2007 that *W. cibaria* was identified as the first bacteriocin-producing strain within the genus, exhibiting inhibitory effects against a few gram-positive bacteria (Srionnual et al., 2007). Furthermore, *W. cibaria* became the center of interest due to its potential antimicrobial and antifungal properties. This eventually raised interest in other *Weissella* strains. In recent times, there have been emerging applications of bacteriocins like weissellin A and bacteriocin D1501 of *W. hellenica* D1501 which have led to the improvement of fermented foods by raising their shelf life (Papagianni, 2012; Chen et al., 2014b; Tenea and Lara, 2019). Moreover, there have been limited reports regarding the mechanism of these antimicrobial substances obtained from *Weissella* spp. (Sturino, 2018).

**Table 3:** Bacteriocins reported from various strains of *Weissella* genus.

**Strain**	**Source**	**Bacteriocin**	**Molecular mass**	**Action against**	**References**
*Weissella confusa* A3	Cow milk	–	2.7 kDa	*Bacillus cereus, Escherichia* *coli, Pseudomonas aeruginosa* and *Micrococcus luteus*	Goh and Philip, 2015
*Weissella hellenica* 4-7	Taiwanese fermented food *sian-sianzih*	Weissellicin L	3.2 kDa	*Listeria monocytogenes*	Leong et al., 2013
*Weissella hellenica* D1501	Chinese traditional Dong fermented meat *Nanx Wudl*	Weissellicin D	62.42 kDa	*Staphylococcus aureus, Listeria* *monocytogenes, Escherichia coli*, yeasts and molds	Chen et al., 2014a,b
*Weissella confusa* LM85	Soil samples	–	–	–	Kaur and Tiwari, 2018
*Weissella cibaria* 110	*plaa-som*, Thai fermented fish product	Weissellicin 110	2.5 kDa	–	Srionnual et al., 2007
*Weissella hellenica* QU 13	Japanese pickles	Weissellicin Y and M	Weissellicin Y- 4924 Da, Weissellicin M-4967 Da	*Bacillus coagulans*	Masuda et al., 2012, 2016
*Weissella cibaria* FMF4B16	Mill flour and fermented cassava	–	–	*Aspergillus niger* MUCL 28699, *Candida albicans* MUCL 30112, *Aspergillus tubingensis* MP1 and *Penicillium crustosum* MY1	Ndagano et al., 2011
*Weissella paramesenteroides* LC11		–	–		
*Weissella paramesenteroides* DFR-8	Cucumber	–	3.74 kDa	*Salmonella typhimurium, Vibrio parahaemolyticus, Aeromonas hydrophila* and *Listeria monocytogenes*	Pal and Ramana, 2010
*Weissella paramesenteroides* DX	Sausage	Weissellin A, class IIa bacteriocin	5 kDa	*Listeria monocytogenes*, *Listeria innocua* and *Clostridium* *sporogenes*	Papagianni and Papamichael, 2011, Papagianni and Sergelidis, 2013
*Weissella cibaria* N23	Thai fermented meat, fish products	–	–	Closely related *Weissella* Strains	Pringsulaka et al., 2012
*Weissella cibaria* KMITL-QU 21	TraditionalThai fermented meat-rice sausage, *Sai-krogIsan*	–	5,975 Da	–	Swetwiwathana et al., 2008
*Weissella hellenica* BCC 7293	Thai fermented pork sausage called *Nham*	Bacteriocins 7293A and 7293B	6.23 and 6.49 kDa	*Pseudomonas aeruginosa, Aeromonas hydrophila, Salmonella Typhimurium* and *Escherichia coli*	Woraprayote et al., 2015
*Weissella confusa* MBF8-1	Indonesian home-made fermented soybean product	Weissellicin- MBF, Bac1, Bac2, and Bac3		*Leuconostoc mesenteroides* and *Micrococcus luteus*	Malik et al., 2021

## 7 Production and purification of *Weissella* bacteriocins

Bacteriocin isolation and purification from *Weissella* spp. was primarily done after obtaining the desired pure colony and optimization of culture conditions for maximum production (Fusco et al., 2015; Goh and Philip, 2015). It was demonstrated that *W. hellenica* QU 13 could possess regulated production of Weissellicins Y and M depending on the nutritional conditions, and varying environmental circumstances (Masuda et al., 2016). To study production of Weissellicin L from *W. hellenica* 4-7, GYP (Glucose Yeast Peptone) medium was studied and optical density of the inoculated culture at 600 nm was measured. Qualitatively, bacteriocin activity was further expressed as arbitrary activity units (AU; reciprocal of the highest dilution at which activity was still obtained) (Leong et al., 2013). In order to assess how well various culture mediums facilitate *W. hellenica* BCC 7293 to produce bacteriocin, TGE (Trypton Glucose Extract Broth; 1% tryptone, 1% yeast extract, 1% glucose, 0.2% Tween 80), TSB (Tryptic Soya Broth), MRS, APT (All Purpose Tween 80), CYG (Casein Yeast Glucose; 1.5% casein sodium salt, 1.5% yeast extract, and 1.0% glucose), WYG (Whey Yeast Glucose; 1.5% whey protein isolate, 1.5% yeast extract, and 1.0% glucose) broths were compared for quantitave production of bacteriocin (Woraprayote et al., 2015). Upon incubation, the cell free culture supernatant harvest was taken further for extraction. Moreover, methods of purification have also been further chosen according to the yield obtained after modification in methodologies (Sharma et al., 2019). For instance, changes in concentrations of reagents such as molarities of buffers, precipitating agents, etc. (Cheigh et al., 2002; Fahim et al., 2017). Primarily used culture medium is De Man, Rogosa, and Sharpe broth (MRS), but Luria Bertani (LB) broth was also used sometimes (Goh and Philip, 2015). Enrichment of the media using different supplements has been in trend after surveying the factors leading to less production of the active principle. It has been an efficient solution to move toward an expected yield (Goh and Philip, 2015; Ma et al., 2020). Monitoring of the Colony Forming Units (CFU) and optical density of the bacteria within different time durations has been a major approach to determining the growth pattern of the organism (Loutfi et al., 2020). Apart from the amount of yield, the activity of obtained bacteriocin and further validation of its potency is a significant step in studies. Every method followed has been with respect to the molecular, chemical, and structural properties of the active component. According to the studies, bacteriocins from *Weissella* spp. have been crucial enough for novel research regarding food safety (Delgado et al., 2005). Considering the methods followed till now, the most conventional has been an initial lab-scale production followed by scaled-up production under optimized conditions (Mahrous et al., 2013). Steps involved are the removal of cellular debris by centrifugation; protein precipitation using appropriate salt and solvent; removal of respective salt or solvent by centrifugation or dialysis; removal of excessive precipitating agent; estimation of total protein content, validation of activity in crude sample expected to contain the active protein; ultra-purification of the crude sample to get the purified component and further application based approaches as depicted in Figure 3 (Chen et al., 2014b; Goh and Philip, 2015; Goyal et al., 2018). Maximum bacteriocin production has been recorded in the early stationary phase using MRS, Glucose Yeast Peptone (GYP), or in some cases All Purpose Tween (APT) culture media (Yang et al., 2018).

The precipitation step has been accordingly optimized depending upon the variations in saturation percentages of the salt or solvent. Ammonium sulfate, acetone, ethanol, trichloroacetic acid, trifluoroacetic acid, or isopropanol are commonly used depending on the properties of the targeted component (Goh and Philip, 2015). In some cases, surfactants have also been known to be contributing for studying the stability and growth responses (Goyal et al., 2018; Wang et al., 2018). Removal of excessive components and impurities up to an extent is further done by dialyzing the sample using buffers of appropriate pH and concentration. Moreover, for storage and washing purposes sodium phosphate buffer and Tris-HCl buffer were most commonly used (Banerjee et al., 2013). The next step of purification is done by gel permeation chromatography and further appropriate gel electrophoresis methods like SDS-PAGE, Native-PAGE, or even modified tricine-PAGE for molecular mass characterization. Ultra-purification was achieved using pre-equilibrated Sepharose or Sephadex columns and purity check using methods like HPLC or LCMS as per the requirement (Wu et al., 2015). After the completion of purification, application-based approaches have been used for food-grade development toward food safety and similar processed products (Pal and Ramana, 2010; Papagianni and Papamichael, 2011; Chen et al., 2013; Bancalari et al., 2020).

Advanced identification techniques for bacteriocins involve a combination of biochemical, genetic, and bioinformatic approaches (Pingitore et al., 2007; Durgadevi et al., 2020).

### 7.1 Mass spectrometry

Mass spectrometry (MS) upon the conventional chromatographic method allows the characterization of bacteriocins based on their mass-to-charge ratio (m/z). Matrix-assisted laser desorption/ionization time-of-flight (MALDI-TOF) MS and electrospray ionization (ESI) MS are efficient techniques for bacteriocin analysis, providing information on peptide mass, sequence, and post-translational modifications. With advancements in sequencing technologies, whole-genome sequencing (WGS) has become a powerful tool for identifying bacteriocin genes.

### 7.2 Bioinformatics tools

These tools utilize algorithms for sequence alignment, motif prediction, and structural modeling to annotate bacteriocin genes and predict their biological activity. Genomic analytical tools can then be used to analyze the genomic data and predict potential bacteriocin-encoding genes based on sequence homology, conserved domains, and structural features.

### 7.3 Structural metagenomics

X-ray crystallography, nuclear magnetic resonance (NMR) spectroscopy, and cryo-electron microscopy (cryo-EM) can be used to determine the three-dimensional structure of bacteriocins. Structural studies provide insights into the molecular mechanisms at the genetic level including potential interactions with target cells and membrane permeabilization.

### 7.4 Machine learning and data mining

Machine learning algorithms can be trained on large datasets of known bacteriocins to predict novel candidates based on sequence features, physicochemical properties, and activity spectra. Data mining approaches can be used to uncover pattern and relation among bacteriocin databases. This can further be validated by high-throughput methods, such as microfluidics-based assays or automated liquid handling systems, to evaluate the potency and spectrum of bacteriocins.

## 8 Properties of *Weissella* bacteriocins

Bacteriocins are diverse in terms of environment, structure, and function, although they have an apparent similarity to yeast and paramecium killing factors. Due to such properties, it has been regarded as a potentially sustainable food preservation agent (Ng et al., 2020). Applications of bacteriocins have been tested and are in the process of evaluating and assessing their application as narrow-spectrum antibiotics. They have drawn immense attention with regard to medicine, as they are hands-on at nanomolar concentrations causing no toxicity to humans. Moreover, their specific mechanisms of low propensity and extremely specific activity to cause resistivity are profitable properties. Many of these have been used as prospective therapeutic agents (Mathur et al., 2017). Bacteriocins have been thus used and consequently known to battle skin infections along with oral, gastrointestinal, respiratory, and urogenital tract infections (Hammami et al., 2013; Sidek et al., 2018).

The bacteriocins obtained from the *Weissella* genus display a wide range of physicochemical properties, making them highly diverse, adaptable, and applicable (Lee et al., 2002; Abriouel et al., 2015; Fusco et al., 2015). These bacteriocins possess several recognized properties, including:

### 8.1 Antimicrobial activity

*Weissella* bacteriocins demonstrate antimicrobial activity against various foodborne pathogens and other disease-causing microorganisms. They have shown efficacy in combating bacteria, viruses, fungi, tumors, obesity-related factors, inflammation, and oxidative stress (Meade et al., 2020). The bacteriocins classified under class II are expected to affect plasma and disturb the membrane integrity of pathogens causing cell death by an efflux of requisite cellular metabolites. The thermal stability and acid resistance of this have been a potential aspect at the industrial level for acidic food preservation (Ahmed et al., 2022). *Weissella* sp. GMP12 is able to inhibit *Staphylococcus aureus* ATCC 6,538 with bacteriocin activity of 3,693.60 AU and *Salmonella* spp. 230C with bacteriocin activity 2,254.17. Moreover, *Weissella* sp. GMP12 inhibits *Klebsiella* sp. CK2 with bacteriocin activity is 3,165.51 AU (Yaafi'Al-Hammam et al., 2023).

### 8.2 Stability in acidic environment

*Weissella* bacteriocins exhibit stability under different temperature conditions and pH ranges. Their optimal activity is typically observed at acidic pH levels between 2 and 6 (Adesina and Enerijiofi, 2016). Masuda et al. (2012) reported the effects of pH and temperature on the activities of purified bacteriocins Weissellicins Y and M at an adjusted pH range of 3.0–11.0 incubation temperature 80, 100, and 121°C for 30 min. Weissellicin M has been observed to be significantly more stable against pH and heat than many other bacteriocins reported earlier.

### 8.3 Sensitivity to proteolytic treatments

These bacteriocins are proteolytically degradable, indicating that they are susceptible to enzymatic destruction. They have, however, performed positively to different enzymatic treatments, including lipase, oxidase, catalase, lysozyme, and amylase (Tenea and Lara, 2019).

### 8.4 Probiotic properties

*Weissella* bacteriocins have been linked to probiotic attributes owing to their capacity to maintain functionality within the gastrointestinal tract (GIT) (Sharma et al., 2018). These bacteriocins are deemed harmless and hold promise for advantageous use (Dobson et al., 2012; Yang et al., 2014; Fusco et al., 2015; Todorov et al., 2020). Studies also signify that bacteriocin like substance produced by *Weissella* sp. GMP12 has the potential to be used as a probiotic starter culture in food fermentations (Yaafi'Al-Hammam et al., 2023). All of these characteristics demonstrate the adaptability and potential usefulness of *Weissella* bacteriocins in a stretch of industries, for instance food preservation, disease prevention, and probiotic formulations. Further research and exploration of aforementioned properties can eventually support the evolution of innovative plans of action for utilizing *Weissella* bacteriocins in different industries.

## 9 Applications of *Weissella* bacteriocins

**Figure 2:**
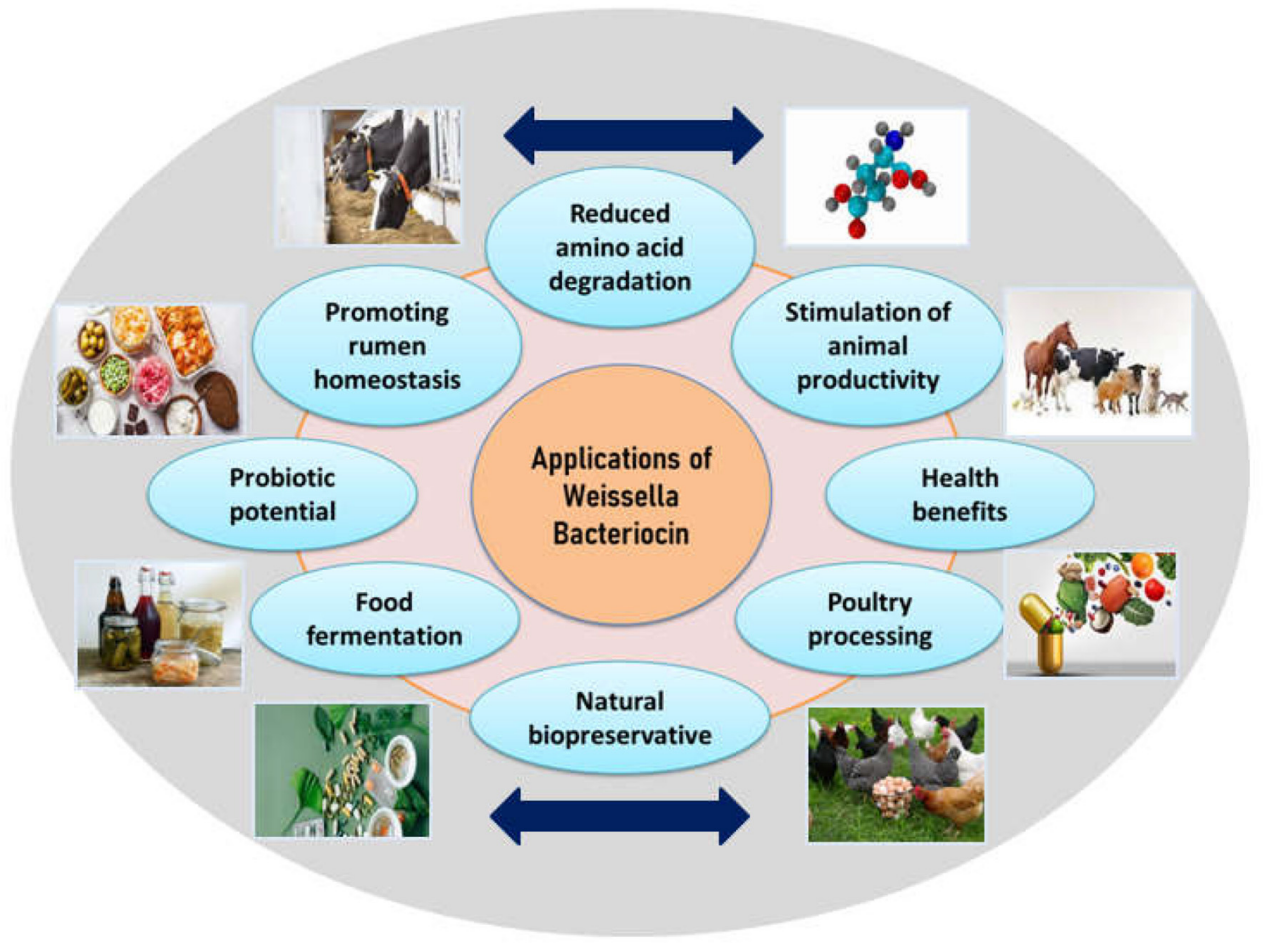
Applications of *Weissella* bacteriocins.

Data about bacteriocin-based therapeutic applications have been independently found in several resources for several years. To sort out this kind of informative deficiency, a basal literature area in BACTIBASE has been developed, which works by grouping papers focusing on bacteriocins and similar substances (Bacteriocin-like substances-BLIS), that have been consequently established as leading therapeutic agents (Newstead et al., 2020). There are reports supporting the potential of bacteriocins for pathogen inhibition in a variety of food matrices such as cheese, meat, and vegetables. Several bacteriocins from validated or notable probiotic bacterial strains have been prospectively evaluated for utilization as potent therapeutic agents for *in-vitro* studies and countable ones for *in-vivo* as well. *W. hellenica* BCC 7239 is known to produce bacteriocins 7293A and 7293B which inhibits pathogens like *P. aeruginosa, E. coli, A. hydrophila and S. Typhimurium* in meat and related products (da Costa et al., 2019). Evaluation of the pharmacokinetics and pharmacodynamics of several bacteriocins proved respective therapeutic efficacies (such as lantibiotic MU1140) (Soltani et al., 2020). Moreover, extensive improvement has been achieved with regard to knowledge about bacteriocin structure and function, regulation, immunity, additional elements affecting cell survival, production of bacteriocin, and its activity thereafter (Meade et al., 2020). Applications of bacteriocins have been reported in various fields as mentioned in Figure 2 and elaborated below:

**Figure 3:**
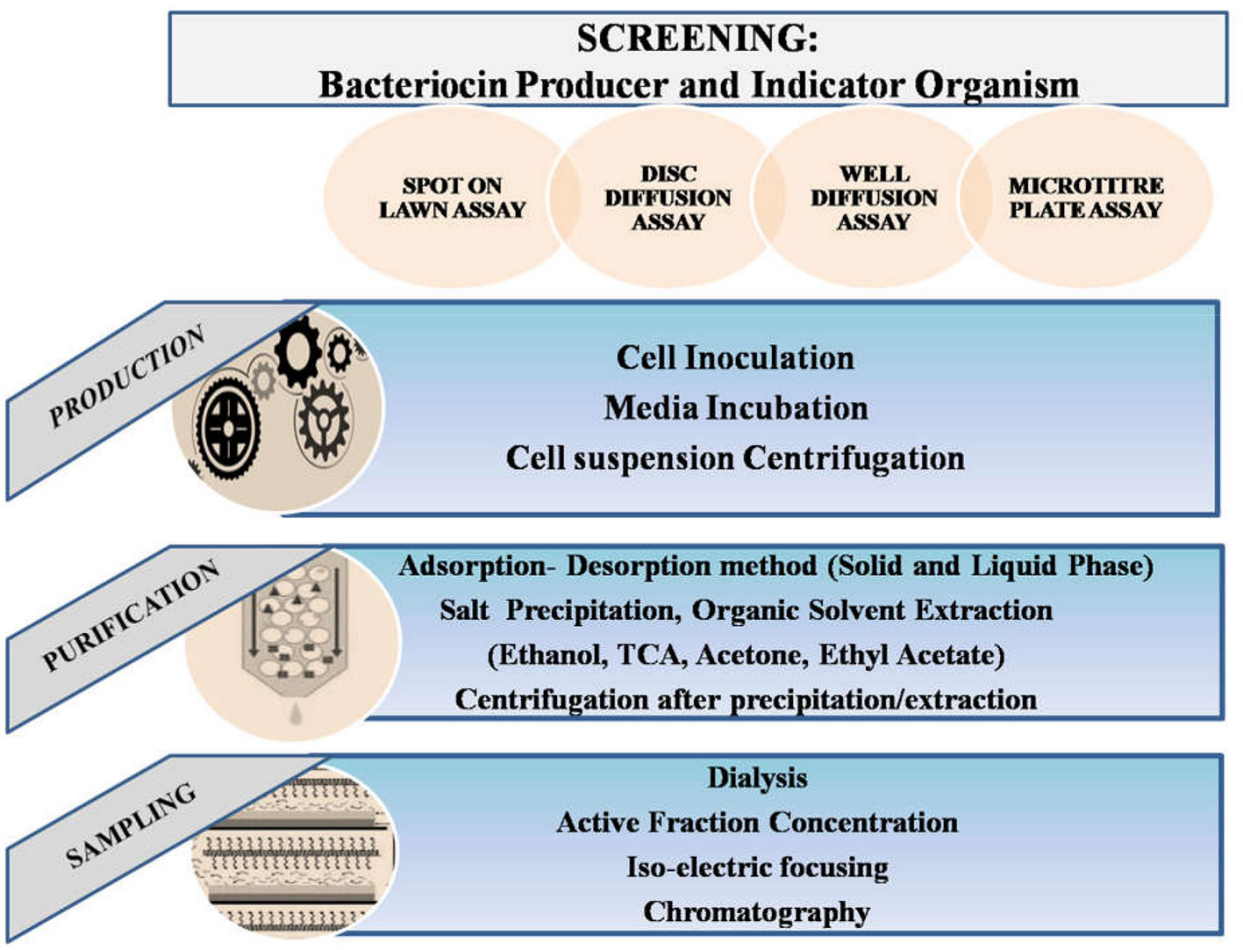
Schemata of bacteriocin production and purification.

### 9.1 Food fermentation

*W. confusa, W. hellenica*, and *W. cibaria* species are studied for some essential properties like the production of extracellular polysaccharides and non-digestible oligosaccharides, which can be further utilized as a probiotic constituent of food, fodder, cosmetics and pharmaceutics (Björkroth et al., 2002; Cole et al., 2006). Bacteriocin A3 produced by *W. confusa* A3 showed strong inhibition against *Micrococcus luteus* ATCC10240, *Escherichia coli* UT181, *Bacillus cereus* ATCC14579, *Enterococcus faecium* C1, *Pseudomonas aeruginosa* PA7, *Lactococcus lactis* A1. This supported the ability of the aforementioned bacteriocins to possess antimicrobial activity against Gram-positive and Gram-negative bacteria as well (Goh and Philip, 2015). Weissellicin D produced by *W. hellenica* D1501 has been reported to inhibit food spoilage bacteria as well as pathogens like *Escherichia coli, Listeria monocytogenes, Staphylococcus aureus*, yeasts, and molds in soymilk (Chen et al., 2014a,b). Weissellicin A from *Weissella paramesenteroides* DX isolated from European-type fermented sausages has been known to be heat resistant and controls the growth of *Listeria monocytogenes, Listeria innocua*, and *Clostridium sporogenes* (Papagianni and Papamichael, 2011; Papagianni and Sergelidis, 2013).

### 9.2 Probiotic application

*Weissella* spp. has also been found to be useful in the form of probiotics, due to their antimicrobial activity. Some of them are *W. cibaria, W. paramesenteroides*, and *W. hellenica* (Fusco et al., 2015). For instance, *W. cibaria is* proposed as an oral probiotic, inhibiting *Streptococcus mutans* which are responsible for glucan biofilm formation (Kang et al., 2009). Weissellicin 110 from *Weissella cibaria* 110 and *Weissella cibaria* KMITL-QU 21 have been known to be acting against potential pathogens (Srionnual et al., 2007; Swetwiwathana et al., 2008). Moreover, bacteriocins from strains like *Weissella cibaria* FMF4B16 and *Weissella cibaria* N23 have been known to inhibit pathogenic bacteria like *Aspergillus niger* MUCL 28699 and related potentially pathogenic *Weissella* spp. strains (Ndagano et al., 2011; Pringsulaka et al., 2012). Also, *W. hellenica* DS-12 from flounder intestine is evidently in use as a fish probiotic, due to its inhibitory activity against pathogens found in fishes, such as *Aeromonas sp., Edwardsiella sp., Pasteurella sp*., and *Vibrio sp*. (Cai et al., 1998). The pro-technological and probiotic potential of *Weissella* spp. therefore strikes off the potential of other human pathogens. Thus, a safety evaluation recommended for every strain which is potentially a starter culture or a probiotic (Ballongue, 2004; Ogier and Serror, 2008).

### 9.3 Stimulation of animal productivity

Bacteriocin-producing bacteria (BPB) have been known to provide many contributions to get beneficial outputs from livestock usage by increasing the productivity of animals. This is obtained by the inhibition of a particular group of organisms (Castillo-González et al., 2014). There are instances to increase feed efficacy, due to the synthesis of bacteriocins by reduction in carbon produced in the form of methane (Rabelo, 2016). The pre-harvest application of BPB for the safety of food is reasoned as one of the vital interventions causing a decrease in the gut-centered colonization of livestock by food-borne pathogenic organisms; for instance, during the processing of feeds like silage, immense contribution has been reported by LAB (Diez-Gonzalez, 2007; Bemena et al., 2014). In this regard, the anti-pathogenic characteristics of *Weissella* bacteriocins against several pathogens could be the prominent option for animal productivity applications (Mantovani et al., 2011).

#### 9.3.1 Promoting poultry processing

Nisin significantly reduced the quantity of Listeria bacteria in scald water from a poultry processing factory, according to Mahadeo and Tatini (1994). There was an instantaneous 2-log (100-fold) decline following therapy, indicating a significant reduction. Moreover, after 48 h of chilling, Listeria was completely eradicated. This proved how effective nisin is at improving food safety while processing poultry. *Salmonella typhimurium* was experimentally contaminated chicken parts' skin. Natrajan and Sheldon (2000a) reported that nisin, EDTA (a chelating agent), and Tween 80 (a surfactant) were administered under acidic conditions via an alginate or agarose-based gel onto the skin of the chicken parts. After 72 h of storage at 4°C, this treatment significantly decreased the pathogen's numbers by up to 3 log10 (1,000-fold). Studies also showed that immersing chilled grill drumsticks in a solution containing nisin could extend their shelf life (Natrajan and Sheldon, 2000b). In addition, they were kept on nisin-treated tray pads and in a Polyvinyl chloride (PVC) overwrap treated with nisin. The chicken pieces' shelf life was increased by 0.6 to 2.2 days using this combined method.

#### 9.3.2 Promoting rumen homeostasis

Bacteriocin, Nisin-based formulation was employed to combat mastitis in lactating cows caused by bacterial pathogens like *Staphylococcus aureus, Streptococcus uberis*, and *Streptococcus dysgalactiae* (Cao et al., 2007; Wu et al., 2007; Field et al., 2021). BPB that produces bacteriocins can help stimulate animal productivity. By inhibiting specific groups of harmful organisms, they create a more favorable environment for livestock, promoting better health and growth. This can result in increased meat and milk production, which is essential for the livestock industry (Bemena et al., 2014; Bennett et al., 2022). Some BPB can synthesize bacteriocins that target methanogenic bacteria, which are responsible for producing methane during the digestive process in the stomachs of ruminant animals. By reducing the populations of these methane-producing bacteria, BPB can reduce methane emissions from livestock, which is a potent greenhouse gas and a significant contributor to climate change, thereby environmentally beneficial. Additionally, reduced methane production may lead to improved feed efficiency, as less energy is lost in the form of methane (Moumen et al., 2016).

#### 9.3.3 Human health benefits

Weissellicin Y, Weissellicin M, and Weissellicin L bacteriocins derived from Weissella strains have been found to have specific inhibitory abilities against *Listeria monocytogenes* ATCC 19111. This suggests their potential use in food safety and preservation to prevent the growth of Listeria bacteria that cause foodborne illnesses. *Weissella* strains have been shown to exhibit chemo-preventive and anti-tumor effects. This suggests their potential use in cancer prevention and treatment (Park et al., 2012; Kwak et al., 2014). Bacteriocins which include compounds like Weissellicin Y and M are reported to have antiviral properties. This has led to their consideration for pharmaceutical and food-based applications. Their high margin of safety makes them attractive for these purposes. Additionally, addressing viral resistance is an ongoing concern, and the use of bacteriocins could help tackle this issue. Reports suggest antiviral activity of bacteriocin enterocin CRL35, obtained from *Enterococcus faecium* CRL35 33 was found to act on the intracellular replication of Herpes Simplex (HSV) type 1 and 2.This was further investigated, suggesting that enterocin CRL35 acts on glycoprotein synthesis of the replication of the virus. Similarly, another study reveals that enterocin ST4V generated by *Enterococcus mundtii* ST4V, inhibited the polio virus by 50% and HSV types 1 and 2 by 99.9% and measles virus by 95%. Synthesis of bacteriocin mutacin 1,140 was associated with *Streptococcus mutans* strain's ability to colonize the oral cavity (Elalem, 2021). *In vitro* studies on *Staphylococcus aureus* demonstrate the potent antibacterial action of mersacidin. Lantibiotics like Pumilicin 4 and haloduracin produced by *Bacillus sphaericus*, have been found to be more stable than nisin at physiological pH values, particularly interesting for medical applications (Malik et al., 2021). There are bacteriocins that can be produced *in vitro* and prove helpful for intestinal tract survival is supported by intestinal bacteria like *Fusobacterium mortiferum* isolated from chicken ceca (Yusuf and Hamid, 2013).

Through their effects on gut microbial communities, bacteriocins may influence the host's immune system in addition to helping the producer survive and colonize in the gut and inhibiting closely related competitor strains or pathogens (Makras and De Vuyst, 2006). When probiotic strains that do not generate bacteriocins are combined with one that does, for example, *Lactobacillus salivarius* strain that produces salivaricin P becomes prevalent in the ileum of pigs (Millette et al., 2008; Walsh et al., 2008; Dobson et al., 2012).

## 10 Conclusion and future perspectives

The review covers the biosynthesis, classification, and naming of bacteriocins within LAB, with an emphasis on *Weissella* spp. Their applications are a testament to the multifaceted benefits of agriculture and animal husbandry as well. However, there is a clear research gap in this area because there hasn't been much research done especially on bacteriocins from the Weissella genus. This review seeks to clarify this issue and motivate scientists to focus on future investigation of these *Weissella* bacteriocins, thus broadening their possible applications. The production and purification methods of *Weissella* bacteriocins are discussed, highlighting their unique properties. These bacteriocins have strong antimicrobial activity against various pathogenic bacteria, making them valuable for food preservation and food safety. *Weissella* spp. also has probiotic potential, promoting healthy microbiota in the human gut and enhancing immunity, another crucial role exists in poultry processing. Although probiotic characterization has been validated, future research may involve rigorous testing of *Weissella* bacteriocins to ensure their safety for consumption in both humans and animals, as well as obtaining regulatory approvals for their use in various applications. Approaches may focus on developing *Weissella* based probiotics with enhanced bacteriocin production, efficacy, and stability. Biotechnological advances may lead to the development of efficient production systems for *Weissella* bacteriocins. This could involve the dynamics of genetic engineering to enhance production or the use of fermentation processes to scale up production for commercial applications of bacteriocins. These findings underscore the versatility of these microorganisms and their bioactive compounds in various fields, including food science, medicine, and health promotion.

## Author contributions

JS: Conceptualization, Writing – original draft, Writing – review & editing, Visualization. PD: Writing – original draft, Writing – review & editing, Visualization. GR: Writing – original draft, Writing – review & editing, Supervision. AJ: Writing – original draft, Writing – review & editing. DK: Writing – original draft, Writing – review & editing, Conceptualization, Supervision. PS: Conceptualization, Supervision, Visualization, Writing – original draft, Writing – review & editing.
